# Electrospun (La,Ba)FeO_3_ Nanofibers as Materials for Highly Sensitive VOC Gas Sensors

**DOI:** 10.3390/s25092790

**Published:** 2025-04-28

**Authors:** Vadim Platonov, Nikolai Malinin, Darya Filatova, Ivan Sapkov, Marina Rumyantseva

**Affiliations:** 1 Chemistry Department, Moscow State University, 119991 Moscow, Russia; 2 Faculty of Materials Science, Moscow State University, 119991 Moscow, Russia; 3 Physics Department, Moscow State University, 119991 Moscow, Russia

**Keywords:** Ba-doped LaFeO_3_, perovskites, semiconductor gas sensor, VOCs, DRIFTS, TPD-MS

## Abstract

In this work, we report the synthesis of perovskite-type Ba-doped LaFeO_3_ (La_1−x_Ba_x_FeO_3_, x = 0.00, 0.02, 0.04, and 0.06) nanofibers (NFs) using the electrospinning method. The synthesized La_1−x_Ba_x_FeO_3_ materials have a fibrous structure with an average fiber diameter of 250 nm. The fibers, in turn, consist of smaller crystalline particles of 20–50 nm in size. The sensor properties of La_1−x_Ba_x_FeO_3_ nanofibers were studied when detecting 20 ppm CO, CH_4_, methanol, and acetone in dry air in the temperature range of 50–350 °C. Doping with barium leads to a significant increase in sensor response and a decrease in operating temperature when detecting volatile organic compounds (VOCs). The process of acetone oxidation on the surface of the most sensitive La_0.98_Ba_0.02_FeO_3_ material was studied using in situ diffuse reflectance infrared Fourier transform spectroscopy (DRIFTS) and temperature-programmed desorption in combination with mass spectrometry (TPD-MS). A mechanism for the sensor signal formation is proposed.

## 1. Introduction

Intensive development of chemical and metallurgical industries, use of power plants operating on biofuel, and high-temperature waste processing and combustion processes lead to an increase in environmental pollution. To control air quality in residential and industrial areas, it is necessary to create selective, stable, and highly sensitive gas sensors. Traditional semiconductor gas sensors based on nanocrystalline oxides (MO_x_ = ZnO, SnO_2_, WO_3_, In_2_O_3_) have a number of serious drawbacks for the quantitative determination of the concentration of toxic impurities in the air, primarily due to low selectivity and drift of parameters caused by low stability of the microstructure during long-term operation at a temperature of 300–500 °C, which is necessary for the detection of reducing gases, including volatile organic compounds (VOCs) [1,2,3,4]. The factors responsible for the instability of the parameters of a semiconductor gas sensor can be classified as follows [5]: (i) structural changes in microheaters and electrodes; (ii) changes caused by processes in the layer of the sensitive material; (iii) changes caused by the processes occurring at the boundaries between the sensitive layer, substrate, and electrodes; and (iv) changes caused by the interaction of the sensitive layer with the components of the surrounding gas phase. The processes occurring in the layer of the sensitive material include structural and phase transformations, as well as the diffusion of oxygen vacancies and other point defects in the volume of the semiconductor oxide. These processes occur with greater intensity under high working temperatures. So, the problem of the stability of the sensor’s characteristics can be solved (at least partially) by creating materials that provide high sensor response at low operating temperatures. Metal oxide perovskites offer tunable electronic properties and the capability for low-temperature operation [6]. Several perovskite materials have been studied as gas sensors due to their stability [7]. Of particular interest as a gas-sensitive material is lanthanum ferrite (LaFeO_3_), a compound belonging to the class of orthoferrites with a distorted orthorhombic perovskite structure. Lanthanum ferrite is a *p*-type semiconductor with a band gap of 2.1–2.6 eV.

Due to its crystal structure, LaFeO_3_ has the ability to substitute atoms in A and B positions within one structural type that provides ample opportunities for creating materials with controlled chemical, electrophysical, sensor, and catalytic properties [8]. The nature of the cations occupying the A and B positions has a very important effect on the electronic structure of materials. The electrostatic potential created by a given cation and the hybridization of its orbitals with the orbitals of the nearest ions affect the width of the energy bands and their arrangement on the energy diagram. In addition, the size of the A cation affects the degree of distortion of the perovskite structure [9]. For this reason, the cationic composition of the A-position also has an important effect on the main properties of perovskite materials (electrophysical, chemical, sensor, and catalytic properties). Based on the available literature data, heterosubstitution of La^3+^ with alkaline earth metal cations (Ba^2+^, Sr^2+^, Ca^2+^) as well as Mg^2+^ and Pb^2+^ cations can increase the sensitivity of the sensor material to volatile organic compounds (VOCs) [10,11,12,13,14,15,16,17] when detecting carbon monoxide [18], sulfur dioxide (SO_2_) [19], carbon dioxide (CO_2_) [20,21,22,23]. Thus, changing the chemical composition of the A-position of perovskite can improve the sensitivity of materials to many analyte gases due to a change in the base resistance of the compounds and the appearance of various point defects (oxygen vacancies, a change in the population of A-positions, a change in the oxidation state of the B-cation) capable of entering into physicochemical interactions with gases in the environment.

The morphology of the LaFeO_3_-based material is another key factor influencing sensor characteristics. Of considerable interest is the production of perovskite oxides with unique morphology and improved properties using new synthesis methods [24]. Gas-sensitive materials based on nanofibers/nanorods/nanowires/nanotubes/etc. can provide high gas permeability even with a small crystallite size and a high surface-to-volume ratio, making them promising for creating gas sensors [25]. The most widely used methods for obtaining nanocrystalline semiconductor oxides are co-precipitation or separate chemical precipitation from aqueous solutions with subsequent annealing in the temperature range of 300–500 °C. The main disadvantage of such methods is the formation of a surface hydrate-hydroxyl layer, which affects the sintering process during the formation of sensitive porous layers and the sensor properties of materials. An alternative is the electrospinning (electroforming) method—the process of forming nanofibers from an electrically charged polymer solution or melt under the action of electrostatic forces [26]. This method allows for a smaller number of steps from non-aqueous polymer solutions to obtain highly dispersed materials of complex composition in the form of fibers, from which a porous structure is formed during synthesis and thermal annealing. Detection of analyte gases by chemoresistive sensors is associated with processes occurring on the surface of a solid; thus, the availability of the surface for the adsorption of detected gases is an important parameter that directly affects the efficiency of the material. The bimodal pore distribution characteristic for materials synthesized by electrospinning improves their sensor properties due to the formation of numerous branched channels in the sample mass, through which the diffusion of molecules of the analyzed gas and reaction products occurs faster than, for example, in sintered powders. In this regard, electrospinning has become widespread in the synthesis of semiconductor oxide materials [27,28,29,30,31,32,33,34,35,36,37]. Successful synthesis of LaFeO_3_-based nanofibers by electrospinning has been reported [38,39,40]. However, the sensor properties of electrospun lanthanum ferrite have been poorly studied to date [41,42].

In this work, La_1−x_Ba_x_FeO_3_ (x = 0.02, 0.04, and 0.06) nanofibers were synthesized by electrospinning precursor-filled polymer solutions with subsequent heat treatment. The effect of Ba content on the structure, morphology, surface properties, and electrophysical and sensor properties in detecting gases of various chemical natures (CO, CH_4_, methanol, and acetone) was studied. Based on the results obtained by in situ diffuse reflectance infrared Fourier transform spectroscopy (DRIFTS) and temperature-programmed desorption coupled with mass spectrometry (TPD-MS), a mechanism for the multi-stage decomposition of acetone on the surface of the sensor material is proposed.

## 2. Materials and Methods

### 2.1. Materials Synthesis

The following reagents were used in the synthesis: polyvinylpyrrolidone (PVP) (Mw = 1,300,000), lanthanum nitrate (La(NO_3_)_3_ × 6 H_2_O), iron acetylacetonate (Fe(acac)_3_), barium nitrate (Ba(NO_3_)_2_), *N,N*-dimethylformamide (C_3_H_7_NO, DMF), and ethanol (C_2_H_5_OH) (all from Sigma-Aldrich (Saint Louis, MO, USA), analytical pure grade). Nanofibers of pristine LaFeO_3_ and La_1−x_Ba_x_FeO_3_ (x = 0.02, 0.04, and 0.06) were synthesized by electrospinning precursor-filled polymer solutions with subsequent heat treatment.

To obtain pure LaFeO_3_ nanofibers, 1.2987 g of lanthanum nitrate and 1.0595 g of iron acetylacetonate were dissolved in a mixture of ethyl alcohol (25 mL) and dimethylformamide (25 mL). When preparing barium-doped lanthanum ferrite nanofibers, the molar ratio of [La]:[Ba]:[Fe] = (1 − x):x:1 (x = 0.02, 0.04, and 0.06) was selected. After complete dissolution of the cation’s precursors, 9 g of polyvinylpyrrolidone was added and stirred until complete dissolution of the polymer and homogenization of the solution. To effectuate the electrospinning process, the polymer solution was placed in a medical syringe, which was fixed in a syringe infusion pump. The solution was fed at a rate of 1 mL/h through a G21 gauge metal needle with an internal diameter of 510 μm. The polymer fibers were formed on a metal collector under a potential difference of 10–11 kV and at a distance of 120–130 mm between the needle and the collector. The resulting polymer fibers were annealed at 600 °C for 5 h, at a heating rate of 1 K/min. This heat treatment mode was selected based on the data obtained for a series of pure LaFeO_3_ samples annealed at different temperatures [43].

### 2.2. Materials Characterization

The elemental composition of the synthesized materials was studied by X-ray fluorescence microanalysis with full external reflection using a S2 PICOFOX spectrometer (Bruker Nano GmbH, Berlin, Germany). Mo Kα radiation was used to excite X-ray fluorescence. The spectrum set time was 250 s. A 5 µL aliquot of the sample solution was applied to a quartz substrate using a dispenser, dried, and analyzed.

The phase composition of the samples was determined by powder X-ray diffraction (XRD). The diffractograms were recorded at room temperature using a DRON-4-0.7 diffractometer (Burevestnik, St. Petersburg, Russia; λ = 1.54051 Å, CuK_α1+α2_ radiation) in the 20–70° 2θ range with 0.1° step and 1°/min speed. Phase identification was effectuated with the use of PDF-1 and PDF-2 databases. The diffraction patterns were processed using the STOE “WinXPOW” software package (Version 1.06). The size of the coherent scattering region (*d_XRD_*) was calculated from the reflection broadening using the Scherrer formula:

(1) $$d_{XRD}=\frac{k\lambda }{\beta cos\theta },$$

where $d_{XRD}$ is the average size of the coherent scattering region, β is the full width at half maximum (FWHM) of the corresponding diffraction peak, λ is the wavelength of the radiation used, θ is the diffraction angle, and k is the shape coefficient for spherical particles (k = 0.9). β was determined as follows:

(2) $$\beta^{2}=\beta_{sample}^{2}+\beta_{standard}^{2},$$

where $\beta_{sample}$ is the FWHM of the diffraction peak of the sample, and $\beta_{standard}$ = 0.085 is the FWHM of the diffraction peak of the standard with a crystallite size over 200 nm.

The specific surface area was determined by low-temperature nitrogen adsorption using a Chemisorb 2750 apparatus (Micromeritics, Norcross, GA, USA) with a thermal conductivity detector. The BET (Brunauer–Emmett–Teller) model was used to calculate the surface area available for gas adsorption. The powder (~100 mg) was placed in a flowing quartz reactor and annealed in 99.999% He flow at 50 mL/min and 300 °C for 1 h to remove adsorbed impurities. Then, a flow of N_2_/He mixture (30 vol.% N_2_) was passed through the reactor at a rate of 12 mL/min, and the sample was cooled to 77 K.

The microstructure and morphology of the nanofibers were studied by scanning electron microscopy (SEM) using a Zeiss Supra 40 FE-SEM electron microscope (Carl Zeiss, Inc., Oberkochen, Germany) with an intralens secondary electron detector at an accelerating voltage of 10 kV and an aperture of 30 µm.

The sample designation, elemental and phase compositions, as well as the microstructure parameters of the synthesized nanofibers are summarized in Table 1.

The surface composition and the charge state of elements were studied by infrared (IR) and X-ray photoelectron (XPS) spectroscopies. IR-spectra were recorded in the transmission mode in the wavenumbers range of 4000–400 cm^−1^ with a step of 4 cm^−1^ using a Perkin Elmer Frontier spectrometer (Perkin Elmer, Waltham, MA, USA). The survey was carried out with potassium bromide tablets (7 mm diameter) containing 1 mass% of the test sample. The tablets were prepared by carefully grinding KBr together with the sample, followed by pressing into tablets at a pressure of 50 bar.

In situ surface composition studies were carried out by diffuse reflectance IR Fourier transform spectroscopy (DRIFTS) using a DiffusIR set-top box and a heated flow chamber HC900 (Pike Technologies, Cottonwood, WI, USA) enclosed by a KBr window. DRIFT spectra were recorded in the region of 4000–1000 cm^−1^ with 4 cm^−1^ resolution. Powder samples weighing 30 mg were placed in aluminum oxide crucibles (5 mm diameter). Before measurement, the samples were heated in a stream of purified air at 450 °C to remove adsorbed impurities.

Studies of the charge state of the elements were carried out using an Axis Ultra DLD spectrometer (Kratos Analytical, Manchester, UK) with monochromatic Al Kα radiation (hν = 1486.7 eV, 150 W) in a vacuum not lower than 10^−9^ Torr. The charge shift was compensated by the C1s ground state peak with a binding energy of 285 eV. Survey spectra in the range of 1300–0 eV with 0.5 eV step were obtained for all samples. The spectra of the Fe2p, La3d, Ba3d, O1s, and C1s regions were recorded with increments of 0.05 eV. The Unifit 2014 program was used to process the spectra, the background was described using the Shirley method, and the spectra were approximated by mixed Gauss–Lorentz functions.

The adsorption/desorption of acetone and its oxidation products on the surface was analyzed using an MS7–200 mass spectrometer (Atomtyazhmash, St. Petersburg, Russia) equipped with an RGA–200 analyzer (Stanford Research Systems, Sunnyvale, CA, USA) during stepwise heating of a sample placed in a quartz tube with an inner diameter of 10 mm. Before the experiment, the sample was kept at 450 °C for 1 h in a flow of He/O_2_ mixture (99:1 vol.%, 30 mL/min) to remove molecules adsorbed from the air on the surface. After cooling the sample to room temperature, a helium–oxygen mixture containing 200 ppm acetone was passed through the sample, and the temperature was gradually raised to 500 °C.

### 2.3. Gas Sensor Measurements

The electrophysical and sensor properties of the synthesized materials were studied in situ by measuring the electrical conductivity of thick films. Materials in the form of a terpineol-based paste were deposited on Al_2_O_3_ micro hotplates with platinum contacts. After applying the paste, the thick films were dried for 5 h at 50 °C, then heated to 550 °C (2 K/min) and kept at this temperature for 3 h to completely remove the binder. The temperature of the films was controlled via resistance heating of the hotplates, and a heating rate of 2 K/min was necessary to prevent destruction of the films due to the difference in the thermal expansion coefficients of the ceramic substrate and deposited material. Three sensors were made from each material.

Measurements of the electrical conductivity of the fibers were carried out in DC stabilized voltage mode using an automated flow cell (PTFE, 100 mL). An RRG-12 electronic gas flowmeter (Eltochpribor, Zelenograd, Russia) was used to create stable flows and obtain gas mixtures with specified concentrations of analyte gases. The IVTM-7 humidity and temperature meter (EXIS, Zelenograd, Russia) was used to control the humidity. The attested gas mixtures were used as sources of analyte gases: 2540 ppm CO in N_2_, 4060 ppm CH_4_ in N_2_, 1020 ppm methanol in N_2_, and 1460 ppm acetone in N_2_. To create gas mixtures containing analyte gases at a given concentration (20 ppm), the attested gas mixtures (from cylinders) were diluted with dry air obtained from a GCHV-2.0-3.5 pure air generator (NPP Chemelectronics, Moscow, Russia). The same dry air was used as the reference gas. The selected concentration (20 ppm) was close to the maximum permissible concentration of the working area for CO (20 ppm) and methanol (15 ppm). In order to be able to compare the sensor signal during the detection of acetone and methane, it was decided to use the same concentration of 20 ppm.

In all experiments, the gas flow through the cell was 100 mL/min. During measurement, the resistance of the sensors was recorded depending on the temperature of the sensor material and the composition of the gas mixture. The sensor response *S* was calculated as follows:

(3) $$S=\frac{R_{gas}-R_{air}}{R_{air}}=\frac{R_{gas}}{R_{air}}-1,$$

where $R_{air}$ is the resistance of the material in the background air, and $R_{gas}$ is the resistance of the material in the presence of the target gas.

## 3. Results and Discussion

### 3.1. Morphology, Microstructure, Elemental and Phase Composition

**Figure 1:**
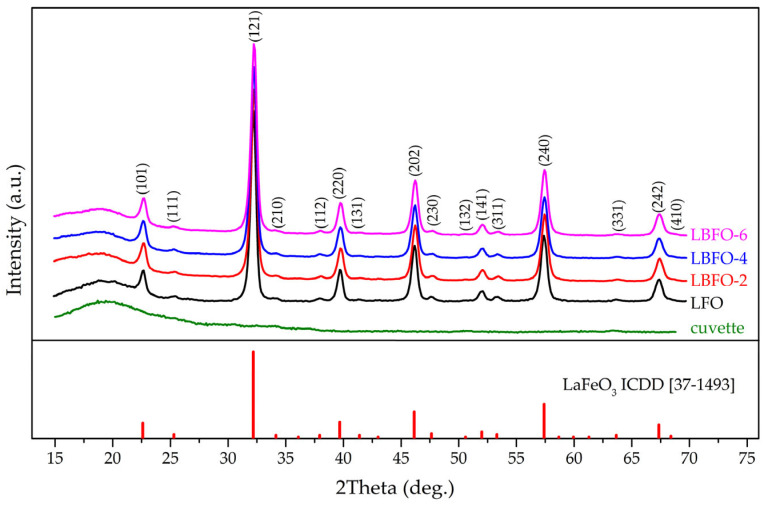
XRD patterns of pure LaFeO_3_ and La_1−x_Ba_x_FeO_3_ series with different Ba contents.

Using X-ray powder diffraction, it was shown that all materials from this series were single phase. The XRD patterns corresponded to LaFeO_3_ with an orthorhombic crystal structure (ICDD card [37-1493]) (Figure 1). The absence of any extraneous reflections indicated the successful incorporation of Ba^2+^ into the La^3+^ sites, which was enabled by the similarity of the effective ion radii between Ba^2+^ (1.61 Å) and La^3+^ (1.36 Å), all of which were in a cuboctahedral environment (coordination number CN = 12). In turn, the introduction of Ba^2+^ into the perovskite B-sublattice was excluded from geometric considerations due to significant discrepancies between the effective radii of these ions (1.35 Å for Ba^2+^) compared to HS Fe^3+^ (0.645 Å) in an octahedral site (CN = 6).

The calculation of the Goldschmidt tolerance factor was carried out according to the formula:

(4) $$t=\frac{r_{A}+r_{O}}{\sqrt{2}*(r_{B}+r_{O})},$$

where $r_{A}$, $r_{B},$ and $r_{O}$ are the effective ionic radii of atoms A, B, and O, respectively, which are part of the perovskite composition ABO_3_. For oxygen in an octahedral environment, $r_{O}$ = 1.4.

In the case of pure LaFeO_3_, the tolerance factor was *t* = 0.95. For the limiting composition of BaFeO_3_, *t* was approximately 1.04, which confirmed the possibility of heterovalent substitution of La^3+^ cations by Ba^2+^ ions at the A-position within the same structural type (perovskite 0.85 < *t* < 1.11) [44]. At the same time, according to the XRD data, the perovskite lattice was distorted and had orthorhombic symmetry with the *Pnma* space group, while ideal perovskites had a primitive cubic structure ($Pm\bar{3}m$ space group). This may be due to oxygen vacancies, the concentration of which may increase because of heterovalent substitution to maintain electroneutrality [14,18,21,22]. Lattice distortion can occur due to another factors, such as changes in the relative positions of the octahedral units relative to each other (e.g., swing-rotation), as well as compression and stretching of these octahedrons [45,46]. The presence of distortions in the perovskite structure after doping was also indicated by a slight shift in the diffraction peaks toward larger angles, despite the larger ionic radius of Ba^2+^ compared to La^3+^. As the dopant concentration increased, the distance between planes tended to decrease, which can be explained by nonstoichiometric oxygen and the inclination of octahedra BO_6_ [47].

The specific surface area of the materials, determined by the low-temperature nitrogen adsorption method using the BET model, was 2–5 m^2^/g. In turn, the introduction of barium into lanthanum ferrite led to a decrease in the crystallite size (Table 1).

**Figure 2:**
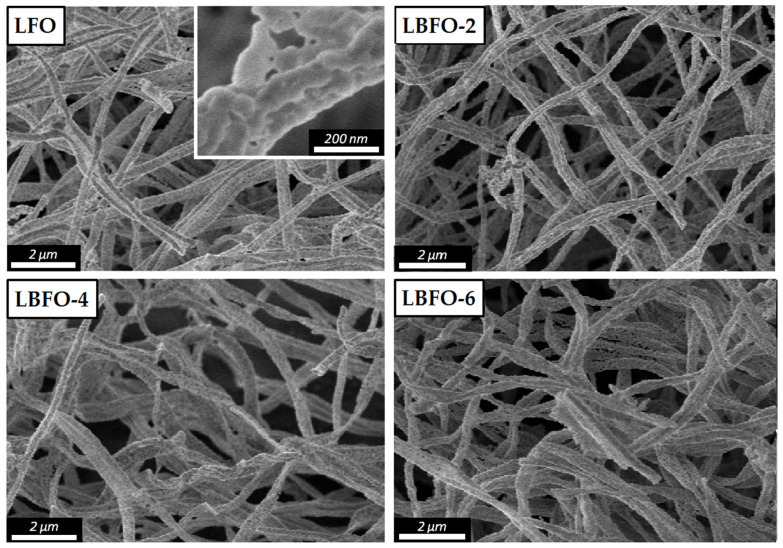
SEM images of pure LaFeO_3_ and La_1−x_Ba_x_FeO_3_ series with different Ba contents.

The morphology of the La_1−x_Ba_x_FeO_3_ samples was investigated using scanning electron microscopy (Figure 2). The samples exhibited a fibrous structure, with an average fiber diameter of 250 ± 20 nm. The fibers, in turn, consisted of smaller crystalline particles with a size of 20–50 nm. Therefore, it could be inferred that the doping process did not significantly alter the macroscopic structure of the materials, which was mostly determined by the synthesis parameters (needle diameter, distance between the needle and collector, voltage, etc.).

### 3.2. Surface Properties

**Figure 3:**
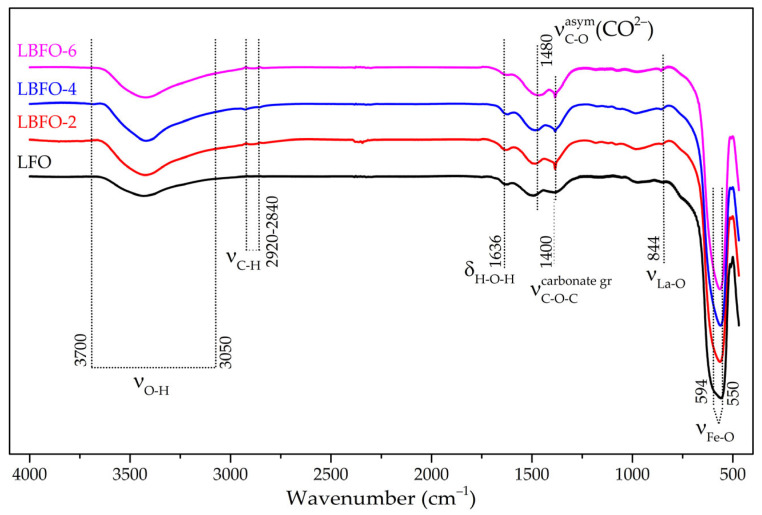
FTIR spectra of pure LaFeO_3_ and La_1−x_Ba_x_FeO_3_ series with different Ba contents.

The analysis of the surface composition of La_1−x_Ba_x_FeO_3_ fibers was carried out using the IR spectroscopy method (Figure 3). The IR spectra of all of the studied samples contained the same set of vibration bands. The broad band at 3700–3050 cm^−1^ and the band at 1635 cm^−1^ were associated with the stretching vibrations of various hydroxyl groups and the deformation vibrations of water molecules adsorbed from atmospheric air. The vibration bands in the range of 2920–2840 cm^−1^ corresponded to C–H bonds, the presence of which was associated with the remains of organic molecules used in the synthesis of LaFeO_3_ nanofibers. The presence of bands at 1480 cm^−1^ and 1400 cm^−1^ was associated with the formation of surface carbonates during annealing, as well as during the adsorption of CO_2_ from air [48]. The band at 844 cm^−1^ was attributed to vibrations of the La–O bond [48]. For an ideal cubic perovskite, the optically active internal vibrations can be classified as Γ_vib_ = 3 F1u + F2u; of which the F1u modes are IR active, while the F2u modes are inactive. These vibrations can be roughly described as follows: the v1(F1u) mode is the B–O stretching vibration of BO_6_ octahedra; the v2(F1u) mode is the deformation vibration of the OBO angle, weakly coupled to the vibrations of the A–O bond; the v3(F1u) mode is associated with the vibration of the A sublattice relative to the BO_6_ octahedra; and the inactive v4(F2u) mode is also associated with deformation vibrations of the OBO angle [49,50]. The expected order of the bands was v1 > v3 > v4 > v2. Since the v4 mode was inactive and the v2 mode was at very low frequencies, the infrared spectrum of cubic perovskite should have two bands. In the case of distorted and low-symmetry perovskites, the v1(F1u) mode may split. Thus, in the recorded spectra (Figure 3), the bands at 594 and 550 cm^−1^ were attributed to vibrations of the Fe–O bonds in the octahedral FeO_6_ groups, corresponding to the v1(F1u) mode [51]. The observed splitting, which appeared as an additional band in the spectrum of the pure lanthanum ferrite sample and as a shoulder in the spectra of barium-doped samples, was apparently due to the distorted crystalline structure. This statement was supported by the X-ray diffraction results, according to which the materials under study had an orthorhombic crystal structure.

**Figure 4:**
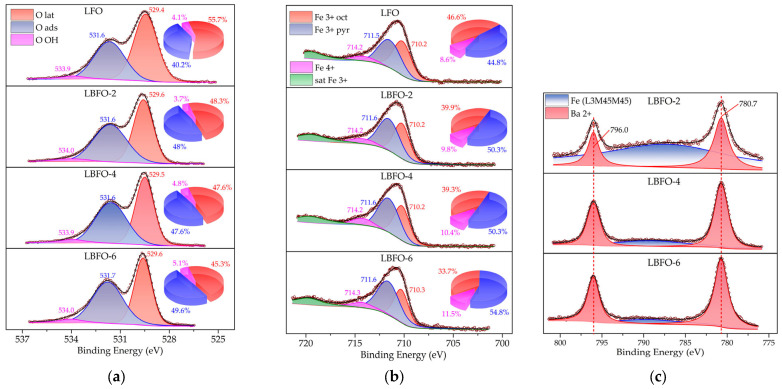
XP-spectra in O1s (**a**), Fe2p (**b**), and Ba3d (**c**) regions of pure LaFeO_3_ and La_1−x_Ba_x_FeO_3_ series with different Ba contents.

Using the method of X-ray photoelectron spectroscopy, we determined the charge state of the elements in the samples. Among them, the spectra of O1s, Fe2p, and Ba3d were the most informative. In the oxygen spectra (Figure 4a), three main components could be distinguished, corresponding to lattice oxygen, adsorbed oxygen, and oxygen in the surface hydroxyl groups. The calculation of the proportions of each component in the spectra showed that the proportion of adsorbed components increased for Ba-doped materials. This indirectly indicated an increase in the number of oxygen vacancies in the LaFeO_3_ structure with the introduction of Ba^2+^. Due to the fact that the oxidation of reducing gases by the Mars–van Krevelen mechanism [52] is unlikely at low temperatures, an increase in adsorbed oxygen may contribute to improving the sensor properties of the doped materials through reactions by Langmuir–Hinshelwood [53] or Eley–Rideal [54] mechanisms.

Because of the complexity of describing the Fe2p region resulting from the various Fe charge states, it was decided to describe the experimental spectrum with the minimum possible number of components (Figure 4b). The most intense components were attributed to Fe^3+^, located in different coordination environments, while the remaining component, located at high energies, was attributed to Fe^4+^ [17,18]. Based on this, it could be concluded that, as the amount of dopant increased, the ratio of Fe^4+^ to Fe^3+^ also increased.

The Ba3d region (Figure 4c) showed that barium had a single charge state of Ba^2+^ on the surface. This, together with the XRD data, allowed us to exclude the possibility of a separate barium-containing phase forming on the grain surface of lanthanum ferrite’s main phase.

Based on an analysis of the literature data, it is known that charge compensation during the doping of LaFeO_3_ with Ba^2+^ ions can occur through the following mechanisms [14,18,21,22]:

(a) Formation of ${\mathrm{Fe}}^{4+}$ ions, ${\mathrm{La}}_{1-x}^{3+}{\mathrm{Ba}}_{x}^{2+}{\mathrm{Fe}}_{1-x}^{3+}{\mathrm{Fe}}_{x}^{4+}O_{3}$;
(b) Oxygen nonstoichiometry, ${\mathrm{La}}_{1-x}^{3+}{\mathrm{Ba}}_{x}^{2+}\mathrm{Fe}O_{3-x/2}$;
(c) Combination of mechanism (a) and (b), ${\mathrm{La}}_{1-x}^{3+}{\mathrm{Ba}}_{x}^{2+}{\mathrm{Fe}}_{1-y}^{3+}{\mathrm{Fe}}_{y}^{4+}O_{3-(x-y)/2}$.

An increase in the amount of oxygen vacancies and of Fe in a higher charge state indicated the possibility of charge compensation through the combination of oxygen nonstoichiometry and Fe^4+^ formation.

### 3.3. Electrical and Gas Sensor Properties

**Figure 5:**
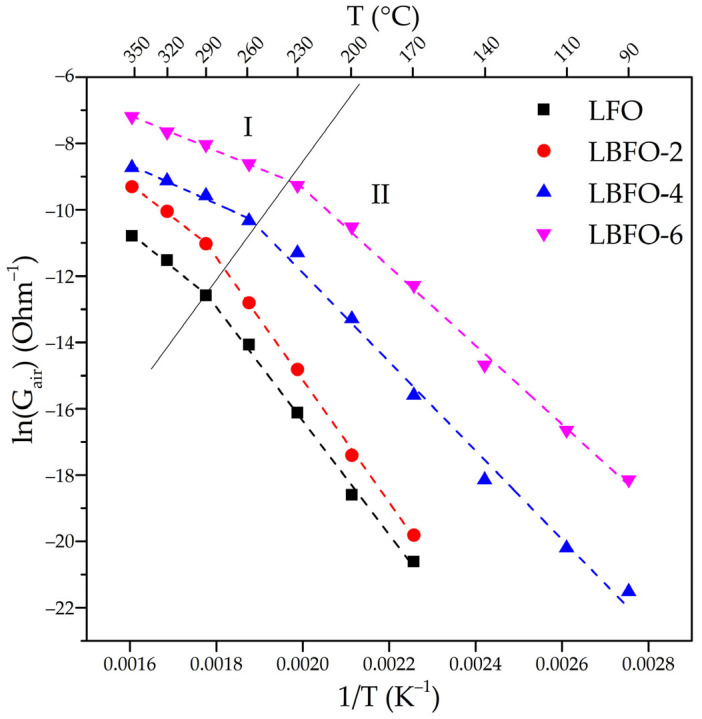
Temperature dependence of the conductivity of pure LaFeO_3_ and materials with different Ba contents.

Measuring the temperature dependence of the materials’ resistance in dry air showed that, with an increase in the dopant content, the base resistance decreased (Figure 5). This can be explained by an increase in the concentration of free charge carriers in a *p*-type semiconductor during heterovalent substitution, according to the quasi-chemical equation below (Wink–Kröger notation):

(5) $${Ba}_{La}^{\times }\to {Ba}_{La}^{\prime }+h^{\cdot }$$

The temperature growth leads to a decrease in the base resistance, which is typical for semiconductors. It follows from Figure 5 that the temperature dependence of conductivity, plotted in Arrhenius coordinates (lnG vs. 1/T), was divided into two regions with different activation energies (Table 2). The low-temperature region (region II in Figure 5) could be attributed to the conductivity associated with the ionization of acceptor levels formed with Ba doping. Conductivity in the high-temperature region (region I in Figure 5) can be described using the small polaron hopping (SPH) mechanism. The introduction of barium into lanthanum ferrite can be considered as the dissolution of BaFeO_3_ in LaFeO_3_ [55]:

(6) $$BaFeO_{3}\overset{LaFeO_{3}}{\to }{Ba}_{La}^{\prime }+{Fe}_{Fe}^{\cdot }+{3O}_{O}^{\times },$$

This leads to the formation of ${Fe}_{Fe}^{\cdot }$. The defect equilibrium is ensured by the incorporation of oxygen into the perovskite matrix, leading to the disappearance of oxygen vacancies and the formation of Fe^4+^ cations:

(7) $$V_{O}^{\cdot \cdot }+{2Fe}_{Fe}^{\times }+\frac{1}{2}O_{2}↔O_{O}^{\times }+{2Fe}_{Fe}^{\cdot }.$$

Charge disproportionation is also possible:

(8) $${2Fe}_{Fe}^{\times }↔{Fe}_{Fe}^{\cdot }+{Fe}_{Fe}^{\prime }.$$

**Table 2:** Activation energies of the conductivity of pure LaFeO_3_ and materials with different Ba contents in high- (E_aI_) and low-temperature (E_aII_) regions (Figure 5). T_k_ denotes the kink temperature on the lnG vs. 1/T dependences (Figure 5).

Sample	E_aI_, eV	E_aII_, eV	T_k_, °C
LFO	0.91	1.48	290
LBFO-2	0.87	1.59	290
LBFO-4	0.50	1.16	260
LBFO-6	0.46	1.02	230

Within the SPH mechanism, it is assumed that the electronic conductivity in ferrites with a perovskite structure occurs via the path $\cdots {Fe}^{4+}-O-{Fe}^{3+}\cdots$ [56]. The degree of overlap of the O2p and the Fe3d orbitals depends on the distance and angle between the iron and oxygen atoms, that is, on the local symmetry of the iron cations. Thus, the distortion of the perovskite crystal structure affects the electronic conductivity realized by the SPH mechanism. At low concentrations of acceptor impurity (x ≤ 0.2 in the La_1−x_Sr_x_FeO_3-δ_ system), electron charge compensation, which is realized by changing the degree of oxidation of iron from 3+ to 4+, occurs predominantly [57]. Since the ionic radii of Fe^4+^ (0.585 Å) and Fe^3+^ (0.645 Å) differ significantly, distortion of the crystal structure is very likely. In this regard, the binding energy of the hole with the iron cation in the polaron may decrease, resulting in a lower activation energy of SPH conductivity and a decrease in the kink temperature (T_k_) on the lnG vs. 1/T dependences (Table 2).

**Figure 6:**
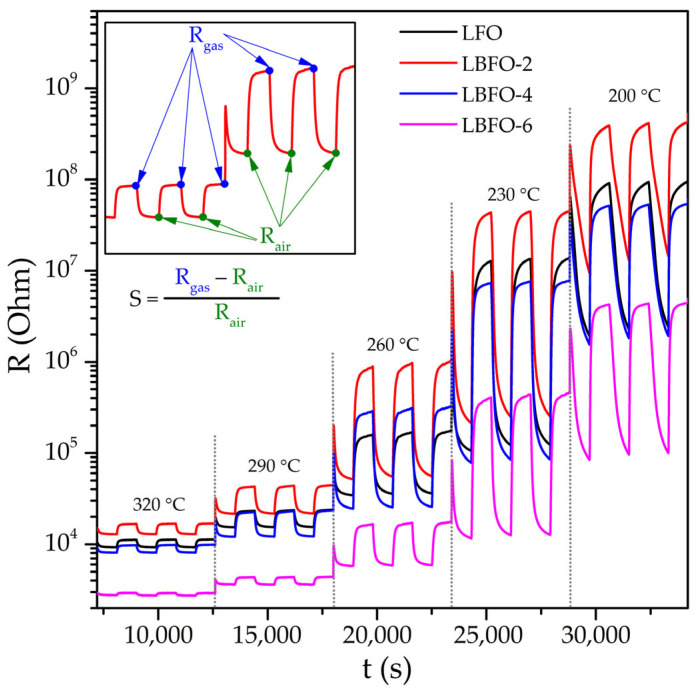
Dynamic change in the resistance of pure LaFeO_3_ and materials with different Ba contents when detecting 20 ppm acetone in dry air at different working temperatures.

The sensor properties of LaFeO_3_ nanofibers were studied when detecting CO, CH_4_, methanol, and acetone. The concentration of all analytes in the gas phase was 20 ppm. The measurements were performed in the mode of periodic change of the gas phase composition (15 min of clean air, 15 min of air with a pollutant gas) in the temperature range of 50–350 °C. Figure 6 shows the dynamic change in the resistance of pure LaFeO_3_ and materials with different Ba contents when detecting 20 ppm acetone in dry air at different operating temperatures. The figure shows five stages of measurements effectuated sequentially at temperatures of 320, 290, 260, 230, and 200 °C. At each stage, three injections of a gas mixture containing acetone were carried out in the sensor cell, followed by purging with clean air at the same temperature. In the presence of the reducing gas, the resistance of the samples increased, corresponding to the *p*-type conductivity of LaFeO_3_. When the sensor chamber was blown with pure air, the resistance of the materials decreased and reproducibly reached the initial value. As the detection temperature decreased, the base resistance of the sensors increased in pure air, reflecting the semiconductor nature of the materials.

**Figure 7:**
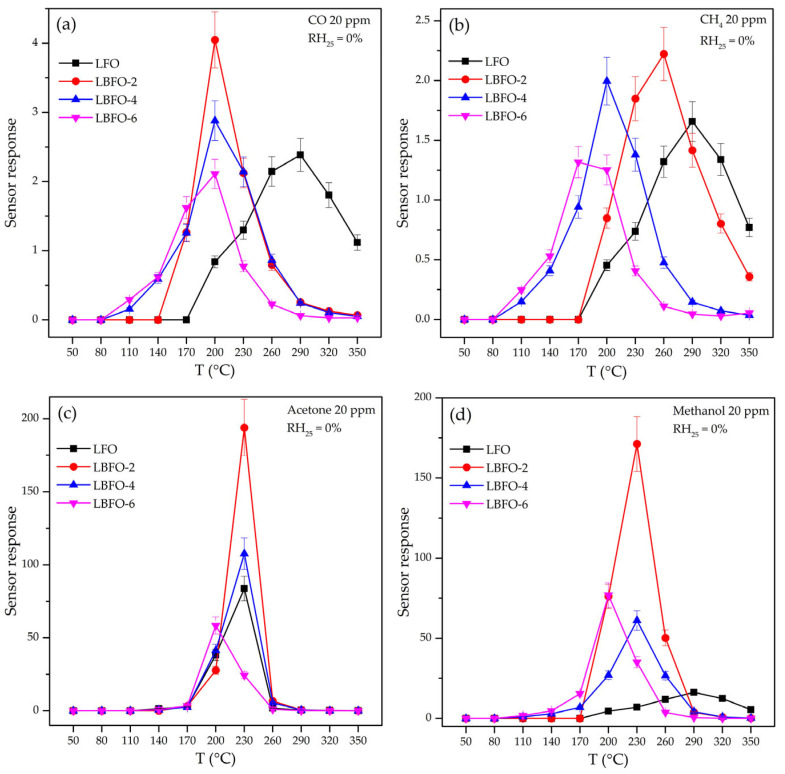
Temperature dependence of the sensor response (S) of La_1−x_Ba_x_FeO_3_ series with different Ba contents toward 20 ppm CO (**a**), CH_4_ (**b**), acetone (**c**), and methanol (**d**).

The temperature dependences of the sensor signal toward 20 ppm CO, CH_4_, methanol, and acetone are shown in Figure 7. The formation of the sensor response of semiconductor oxide-based materials was due to the oxidation reaction of the analyte gases by oxygen chemisorbed on the surface:

(9) $${CO}_{\left(gas\right)}+\frac{1}{m}O_{m\;(ads)}^{n-}\to {CO}_{2\;(gas)}+\frac{n}{m}e^{-},.$$

(10) $${CH}_{4\;(gas)}+\frac{4}{m}O_{m\;(ads)}^{n-}\to {2H}_{2}O_{\left(gas\right)}+{CO}_{2\;(gas)}+\frac{4n}{m}e^{-},$$

(11) $${CH}_{3}{OH}_{\left(gas\right)}+\frac{3}{m}O_{m\;(ads)}^{n-}\to {CO}_{2\;(gas)}+{2H}_{2}O_{\left(gas\right)}+\frac{3n}{m}e^{-},$$

(12) $${CH}_{3}{C\left(O\right)CH}_{3\;(gas)}+\frac{8}{m}O_{m\;(ads)}^{n-}\to {3CO}_{2\;(gas)}+{2H}_{2}O_{\left(gas\right)}+\frac{8n}{m}e^{-}.$$

where ${CO}_{\left(gas\right)}$, ${CH}_{4(gas)}$, ${CH}_{3}{OH}_{\left(gas\right)}$, and ${CH}_{3}{C\left(O\right)CH}_{3(gas)}$ are molecules in the gas phase; $O_{m(ads)}^{n-}$ is a particle of chemisorbed oxygen (m = 1 or 2, n = 1 or 2, possible options: $O_{2}^{-}{,O}^{-},O^{2-}$); $e^{-}$ is an electron that is injected into the conduction band as a result of the reaction; and ${CO}_{2(gas)}$ and $H_{2}O_{\left(gas\right)}$ are molecules of reaction products desorbed from the surface of the material into the gas phase.

**Figure 8:**
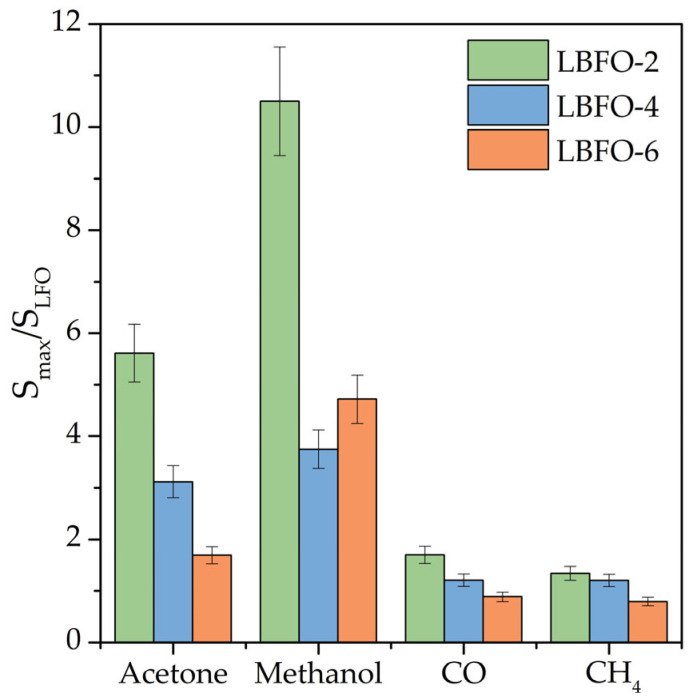
The ratio of the maximum sensor response of barium-doped materials to the response of LaFeO_3_ nanofibers when detecting VOCs, CO, and CH_4_ at appropriate operating temperatures.

The measurement error of the sensor response (based on the reproducibility of data obtained from three sensors made of each material) was no more than 10%. It is worth noting the significantly greater sensor responses of barium-doped and pure LaFeO_3_ when detecting volatile organic compounds compared to CO and CH_4_ (Figure 7). On the one hand, this effect can be explained by the “multi-electron” nature of the oxidation process of VOC molecules on the surface of the sensor material by chemisorbed oxygen (Equations (9)–(12)). The more chemisorbed oxygen molecules are involved in the oxidation of the analyte gas molecule, the more electrons released during the reaction are injected into the conduction band, leading to a greater change in the conductivity of the material and, accordingly, a greater sensor response. On the other hand, this may be due to the high catalytic activity of coordinatively unsaturated iron cations on the La_1−x_Ba_x_FeO_3_ surface. As is known, iron oxides are used as a catalyst for the oxidation of methanol and acetone [58,59,60,61]. In addition, the high sensor responses to acetone and methanol may be due to both the lower dissociation energy of bonds in VOC molecules relative to carbon monoxide and methane [62] (Table 3) and the lower adsorption energy of VOC molecules due to the formation of a bond between the coordinatively unsaturated Fe^n+^ cations and the oxygen of the organic molecule. From the obtained results, it followed that the introduction of barium led to some decrease in the operating temperature (Figure 7) and, in the case of VOCs (methanol and acetone), to a significant increase in the sensor response (Figure 8). The above discussed combined mechanism of charge compensation when replacing La^3+^ cations with Ba^2+^ cations provided an increase in the amount of chemisorbed oxygen and the concentration of Fe^4+^, which, in turn, facilitated the oxidation of VOCs on the surface of barium-doped materials. A comparison of the sensor responses of LaFeO_3_-based materials described in the literature and obtained in this work (Table 4) led to the conclusion that the combination of nanofiber morphology and barium doping made it possible to form materials that provide a high sensor response to acetone at a reduced operating temperature.

**Figure 9:**
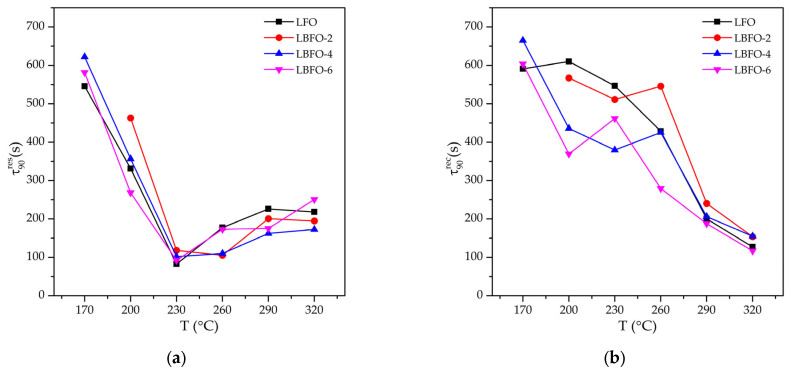
The temperature dependences of response time ($\tau_{90}^{res})$ (**a**) and recovery time ${(\tau }_{90}^{rec})$ (**b**) of La_1−x_Ba_x_FeO_3_ nanofibers when detecting acetone.

To assess the prospects of practical use of sensors based on La_1−x_Ba_x_FeO_3_ nanofibers, we estimated the response ($\tau_{90}^{res}$) and recovery $(\tau_{90}^{rec}$) times when detecting acetone (Figure 9). Even though the absolute $\tau_{90}^{res}$ and $\tau_{90}^{rec}$ values are strongly dependent on the parameters of the testing system, they are useful for comparing the characteristics of materials if the measurements are performed under identical conditions. It was noted that the measurement temperature had a major influence on the $\tau_{90}^{res}$ and $\tau_{90}^{rec}$ values, while the composition of the material was of minor importance for the dynamic characteristics. This is not surprising since the morphology of nanofibers (which may be an important parameter determining the transport of gases to the surface of crystallites of a sensitive material) did not change when La^3+^ cations were replaced by Ba^2+^ cations. It should be noted that, at a temperature of 230 °C, which corresponded to the maximum sensor response toward acetone, a minimum response time was observed, while the recovery time was significantly longer. To speed up sensor recovery when operating under real conditions, a two-temperature operation mode can be used, in which the sensor is shortly heated (for several seconds) to a higher temperature (for example, up to 300 °C). This operation mode will not lead to a significant increase in power consumption; however, it will ensure effective desorption of the products of oxidation of the analyte gas from the surface of the sensitive layer.

### 3.4. Model of Forming La_1−x_Ba_x_FeO_3_ Nanofibers’ Sensor Response

It is noteworthy that the temperature range at which the sensor response was observed during VOC detection was quite narrow (170–290 °C), which may be due to a non-trivial mechanism of the oxidation reaction of the VOC molecules on the surface of the sensor material. To establish the reaction pathway during acetone oxidation on the La_1−x_Ba_x_FeO_3_ surface, additional studies were carried out, including in situ IR spectroscopy in the diffuse reflectance mode (DRIFTS) and temperature-programmed desorption of acetone in combination with mass-spectral analysis of desorption products (TPD-MS). The LBFO-2 sample characterized by the maximum sensor response in acetone detection was selected for this study.

**Figure 10:**
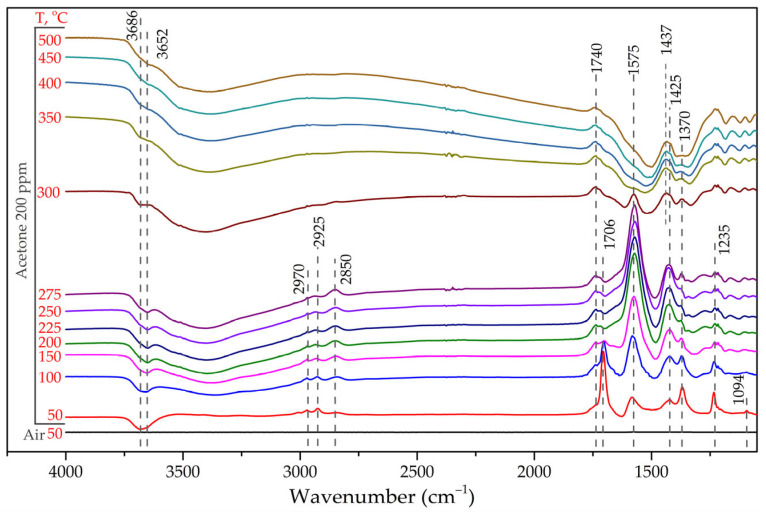
DRIFT spectra of La_0.98_Ba_0.02_FeO_3_ nanofibers (sample LBFO-2) under acetone adsorption (200 ppm in dry air) in the temperature range of 50–500 °C.

Figure 10 shows the evolution of the LBFO-2 DRIFT spectra in the presence of 200 ppm acetone with increasing temperature. After keeping the sample in a flow of acetone at a temperature of 50 °C, bands appeared in the spectra at 1094, 1235, 1370, 1706, 1740, 2925, 2970, 3652, and 3686 cm^−1^, corresponding to vibrations of ω(CH_2_), ν(C–C), δ_s_(CH_3_), ν(C=O), ν(C–O), ν_as_(CH_3_), ν_s_(CH_3_), and ν(O–H) of the acetone molecule adsorbed on the surface, respectively [64,65,66,67]. The spectra also contained low-intensity bands at 1425, 1575, and 2850 cm^−1^, the intensity of which increased significantly with increasing temperature; the intensity of the bands at 1740 and 1370 cm^−1^ did not change; and the intensity of the bands corresponding to acetone decreased sharply. The band at 1425 cm^−1^ may have corresponded to ν_s_(C–O–C); the bands at 1575 and 1370 cm^−1^ were related to ν_as_(COO^–^) and ν_s_(COO^–^), respectively; and the band at 2850 cm^−1^ was related to ν_s_(CH_2_) vibrations in formate groups formed on the surface during acetone oxidation [64,65,68,69,70]. The retention of the intensity of the band at 1740 cm^−1^ may have been related to the formation of carbonates on the surface of the material. At temperatures above 300 °C, the bands corresponding to the vibrations of formate groups disappeared. The signals at 1740 and 1437 cm^−1^ corresponded to vibrations of ν(C–O) and ν_s_(O–C–O) of carbonates formed during the oxidation of formates, respectively.

**Figure 11:**
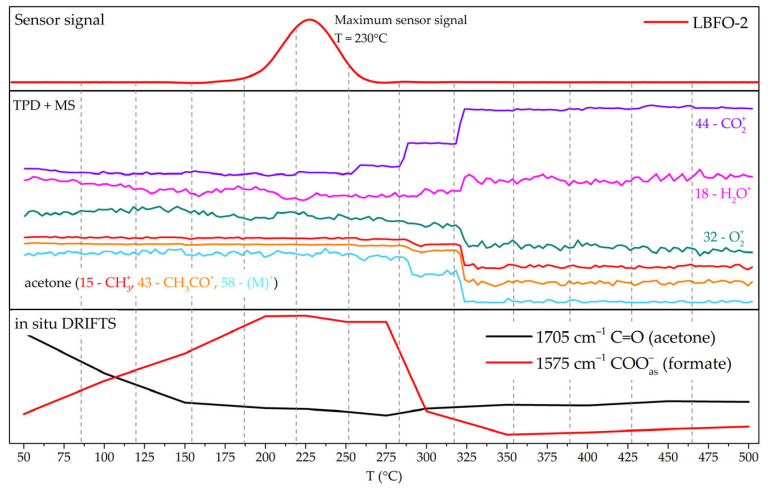
Acetone oxidation on the surface of La_0.98_Ba_0.02_FeO_3_ nanofibers (sample LBFO-2).

At the same time, according to the mass spectra, starting from a temperature of 300 °C, significant increases in the amounts of water and CO_2_ occurred when the sample was purged with acetone (Figure 11). It was assumed that, under these conditions, acetone combustion occurred, which was probably facilitated by Fe ions in the perovskite composition acting as a catalyst.

If we consider the average temperature interval, increasing the temperature up to 250 °C led to the disappearance of carbonyl groups and the appearance of formate groups. The greatest sensor signal was observed in the same temperature interval, indicating that it was the process of acetone oxidation to formate groups and their further decomposition to CO_2_ and H_2_O that included the largest amount of chemisorbed oxygen, which greatly changed the concentration of free charge carriers in the semiconductor.

Based on this, we can propose the following mechanism for the reaction of acetone with the surface of the sensing material and the formation of the sensor response.

At low temperatures, acetone adsorbs on the active centers of the lanthanum ferrite surface and forms formates (region 50–190 °C in Figure 11).

(13) $${CH}_{3}{COCH}_{3(gas)}\to {CH}_{3}{COCH}_{3(ads)},.$$

(14) $${CH}_{3}{COCH}_{3(ads)}+\frac{2}{\beta }O_{\beta (ads)}^{\alpha -}\to {3HCOH}_{\left(ads\right)}+{2\alpha e}^{-}.$$

At temperatures above 250 °C, acetone oxidation occurs mostly due to oxygen from the surrounding air and not due to oxygen chemisorbed on the surface. This assumption was supported by a decrease in the ion current corresponding to oxygen in the mass spectra. It appears that acetone combustion occurs at the surface of the material, forming carbon dioxide and water, without intermediate stages (region 250–500 °C in Figure 11).

(15) $${CH}_{3}{COCH}_{3(gas)}+O_{2(gas)}\overset{{Fe}_{x}O_{y}}{\to }{3CO}_{2\left(gas\right)}+H_{2}O_{\left(gas\right)}.$$

**Figure 12:**
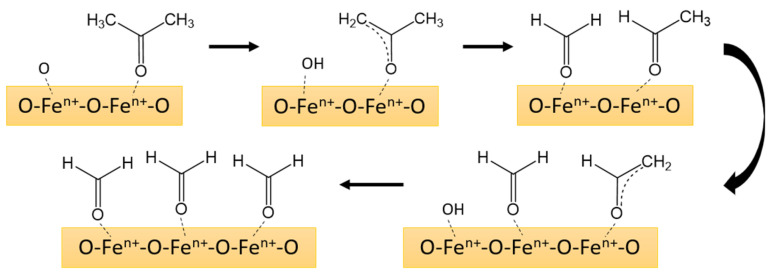
Scheme of step-by-step oxidation of acetone to formate on the La_1−x_Ba_x_FeO_3_ surface.

The processes occurring in the temperature range of 190–250 °C (Figure 11) can be described by the following reactions of multi-stage oxidation of formate to CO_2_ and H_2_O, accompanied by the release of a large number of localized electrons from chemisorbed oxygen [71]. Schematically, the multi-stage process of acetone oxidation on the surface of the sensor material is shown in Figure 12.

(16) $${\beta \cdot HCOH}_{\left(ads\right)}{+O}_{\beta \left(ads\right)}^{\alpha -}\to {\beta \cdot H}_{2}{COO}_{\left(ads\right)}^{-}+(\alpha -1)\cdot e^{-},$$

(17) $${\beta \cdot H}_{2}{COO}_{\left(ads\right)}^{-}{+O}_{\beta \left(ads\right)}^{\alpha -}\to {\beta \cdot HCOO}_{\left(ads\right)}^{2-}+\beta \cdot {OH}_{\left(ads\right)}^{(\alpha -1)-},$$

(18) $${2\beta \cdot HCOO}_{\left(ads\right)}^{2-}{+O}_{\beta \left(ads\right)}^{\alpha -}\to {2\beta \cdot CO}_{2\left(gas\right)}+{\beta \cdot H}_{2}O_{\left(gas\right)}+{(4\beta +\alpha )\cdot e}^{-}$$

## 4. Conclusions

Ba-doped LaFeO_3_ nanofibers (La_1−x_Ba_x_FeO_3_, x = 0.00, 0.02, 0.04, and 0.06) with an average diameter of about 200 nm were obtained by electrospinning. The obtained materials were single phase, had an orthorhombic structure, and consisted of nanocrystallites with a size of about 14–16 nm. The introduction of barium led to the inhibition of crystallite growth during isothermal annealing and promoted an increase in the sensor response of the LaFeO_3_ nanofiber-based sensors toward VOCs. The La_0.98_Ba_0.02_FeO_3_ sample demonstrated the highest sensor response and a decrease in the operating temperature. The improvement in the gas-sensitive properties of the doped materials can be explained by the high catalytic activity of the surface of synthesized materials associated with the formation of oxygen vacancies, highly active iron cations (Fe^4+^), and coordinatively unsaturated cations (Fe^3+^). The mechanism of acetone oxidation on the sensor surface, studied by DRIFTS and TPD-MS methods, is assumed to have a multi-stage nature.

## Figures and Tables

**Table 1:** Sample designations, elemental and phase compositions, and microstructure parameters of synthesized nanofibers.

Sample	Material	[Ba]/([La]+[Ba]) at.%, RSD ^1^ = 4% (XRF)	Phase Composition	d_XRD_, nm
LFO	LaFeO_3_	-	lanthanum ferrite LaFeO_3_ ICDD [37-1493]	17 ± 2
LBFO-2	La_0.98_Ba_0.02_FeO_3_	2		14 ± 1
LBFO-4	La_0.96_Ba_0.04_FeO_3_	4		14 ± 1
LBFO-6	La_0.94_Ba_0.06_FeO_3_	6		14 ± 1

^1^ Relative standard deviation.

**Table 3:** Bond dissociation energies in selected gas molecules [62].

Molecule	Bond	ΔH_dis_, kJ/mol
CO	C≡O	1075
CH_4_	H–CH_3_	431
Acetone	C=O	745
	C–H	414
	C–C	347
Methanol	C–O	384
	C–H	337
	O–H	428

**Table 4:** Summary of the sensor response values toward acetone of LaFeO_3_-based materials described in the literature and obtained in this work.

Material	Synthesis Method	Working Temperature (°C)	Acetone Concentration, ppm	Sensor Response	Ref.
La_0.7_Sr_0.3_FeO_3_	Sol-Gel	275	500	0.7	[10]
(La,Ba)(Fe,Ti)O_3_	Sol-Gel	132	100	19	[11]
La_0.75_Ba_0.25_FeO_3_	Sol-Gel	275	20	2.1	[12]
La_0.75_Ba_0.25_FeO_3_	Sol-Gel	240	500	172	[14]
La_0.98_Ba_0.02_FeO_3_	hydrothermal	200	100	8	[15]
La_0.9_Sr_0.1_FeO_3_	PLD	303	4	0.77	[16]
La_0.98_Mg_0.02_FeO_3_	PMMA template method	190	100	50	[17]
La_0.8_FeO_3_	electrospinning	180	100	6	[63]
La_0.98_Ba_0.02_FeO_3_	electrospinning	230	20	194	This work

## Data Availability

The original contributions presented in this study are included in the article. Further inquiries can be directed to the corresponding author.
